# Preventing physical and chemical degradation of the LABL-Fc-MOG_R5_, a bifunctional peptide inhibitor, with formulation development approaches

**DOI:** 10.1093/abt/tbag005

**Published:** 2026-02-24

**Authors:** Lun Xin, Rucha Mahadik, Monika Prorok, Marcela Solis Rodriguez, Kai Qing Chan, Thomas Tolbert, Yunsong Li, Teruna J Siahaan

**Affiliations:** BioDev Department, WuXi Biologics USA, Cranbury, NJ 08512, United States; Department of Pharmaceutical Chemistry, University of Kansas, Lawrence, KS 66047, United States; Department of Pharmaceutical Chemistry, University of Kansas, Lawrence, KS 66047, United States; BioDev Department, WuXi Biologics USA, Cranbury, NJ 08512, United States; Department of Pharmaceutical Chemistry, University of Kansas, Lawrence, KS 66047, United States; Department of Pharmaceutical Chemistry, University of Kansas, Lawrence, KS 66047, United States; Department of Pharmaceutical Chemistry, University of Kansas, Lawrence, KS 66047, United States; BioDev Department, WuXi Biologics USA, Cranbury, NJ 08512, United States; Department of Pharmaceutical Chemistry, University of Kansas, Lawrence, KS 66047, United States

**Keywords:** primary progressive multiple sclerosis (PPMS), bifunctional peptide inhibitor (BPI), fc-fusion protein stability, high-throughput formulation screening, design of experiment (DoE), protein aggregation, hydroxypropyl-β-cyclodextrin (HP-βCD)

## Abstract

**Background:**

LABL-Fc-MOG_R5_ is a bifunctional peptide inhibitor designed to modulate immune responses in multiple sclerosis, including primary progressive multiple sclerosis. Although therapeutically promising, its development has been hindered by poor physical stability, particularly rapid precipitation under standard formulation conditions.

**Methods:**

A high-throughput, multi-phase formulation strategy was implemented to improve conformational and colloidal stability. An initial Phase 1 evaluation used intrinsic fluorescence differential scanning fluorometry and polyethylene glycol (PEG) solubility assays to screen 96 formulations spanning a range of pH values, buffer systems, and excipients. Then, a Phase 2 study applied a definitive screening design to evaluate key formulation variables—including buffer type, excipients, and surfactant concentration—at reduced protein concentration in 96-well plates. A final Phase 3 study compared the lead formulation from Phase 2 with the control formulation at higher protein concentration in glass vials.

**Results:**

The Phase 1 study revealed pronounced sensitivity of LABL-Fc-MOG_R5_ to pH and ionic strength. Divalent anionic buffers (e.g. citrate, succinate) and excipients such as sucrose and hydroxypropyl-β-cyclodextrin substantially reduced aggregation propensity. In the Phase 2 study, an optimized formulation was identified—10 mM sodium acetate, pH 5.3, 125 mM sucrose, 150 mM HBP-LB-βCD, and 0.025% polysorbate 80—based on statistical modeling. This formulation demonstrated markedly improved resistance to aggregation, fragmentation, and subvisible particle formation under accelerated stress relative to the control.

**Conclusions:**

This work underscores the value of excipient selection and rational design of experiments in stabilizing complex fusion proteins. The optimized formulation provides a significantly enhanced stability profile and supports further development of LABL-Fc-MOG_R5_ toward clinical evaluation for primary progressive multiple sclerosis.

## Teaser

Statement of Significance The clinical advancement of bifunctional peptide inhibitors is often limited by their physical instability in aqueous formulations. This study presents a systematic and data-driven approach to overcome the aggregation and fragmentation challenges associated with LABL-Fc-MOG_R5_, a fusion protein that effectively suppresses experimental autoimmune encephalomyelitis in mice, an animal model for primary progressive multiple sclerosis. By integrating high-throughput screening, definitive screening design, and orthogonal analytical assessments, the study produces a robust, isotonic formulation that preserves protein integrity under thermal, mechanical, and freeze/thaw stress. These findings not only provide a viable pathway for advancing LABL-Fc-MOG_R5_ toward clinical development but also establishes a broader framework for stabilizing structurally similar Fc-fusion therapeutics.

## Introduction

Multiple sclerosis (MS) is a chronic autoimmune disease with characteristic inflammation of the central nervous system; this leads to neural degeneration and demyelination causing neurological disabilities in adults [1, 2]. In the early stages, autoreactive T cells attack the neuronal axon that is coated by specific myelin sheath proteins such as myelin basic protein (MBP), myelin oligodendrocyte glycoprotein (MOG), and proteolipid protein (PLP). The damage in the myelin sheath generates different MS subtypes like relapse-remitting MS (RRMS), secondary progressive MS (SPMS), and PPMS [3, 4]. As the most common form of the disease, RRMS affects 87% of MS patients and is marked by acute attacks followed by remission periods. Over time, ~65% of patients transition to SPMS, which is characterized by irreversible neuron damage. The least common subtype, PPMS, accounts for 5% of cases and progresses without remission, often accompanied by symptoms like eye pain, dizziness, and depression [5].

Developing treatment for MS is challenging due to different disease states being associated with distinct myelin antigens; thus, treatments of MS require tailored approaches [6]. Current options include anti-inflammatory drugs that alleviate symptoms and disease-modifying therapies (DMTs) that alter disease progression by suppressing immune system activity [7]. In a recent development for a new category of DMTs, bifunctional peptide inhibitors (BPIs) target sub-populations of autoreactive T cells that are sensitive to myelin proteins such as PLP, MBP, or MOG. There are three components of BPIs, which consist of an antigenic peptide for signal-1, a spacer, and a signal-2 blocking peptide [8]. This spacer can be aminocaproic acid, polyethylene glycol, or an Fc domain [8]. The hypothesis is that BPI molecules bind to MHC-II and ICAM-1 on the surface of antigen-presenting cells (APCs) to disrupt the immunological synapse (IS) formation when APCs interact with naïve T-cells [4]. As a result, BPIs alter the balance of naïve T cell commitment from inflammatory to regulatory phenotypes [4]. Unfortunately, early BPIs that were linked by aminocaproic acid or polyethylene glycol faced challenges with *in vivo* plasma stability and clearance [9]. Accordingly, Fc-BPIs have been engineered with the IgG1 Fc region functioning as a linker between an antigenic peptide (e.g. MOG, MBP, or PLP) and a signal-2–blocking LABL peptide, resulting in an extended half-life and enhanced efficacy in suppressing experimental autoimmune encephalomyelitis (EAE) in mice compared to their parent BPIs [4, 10, 11].

**Figure 1:**
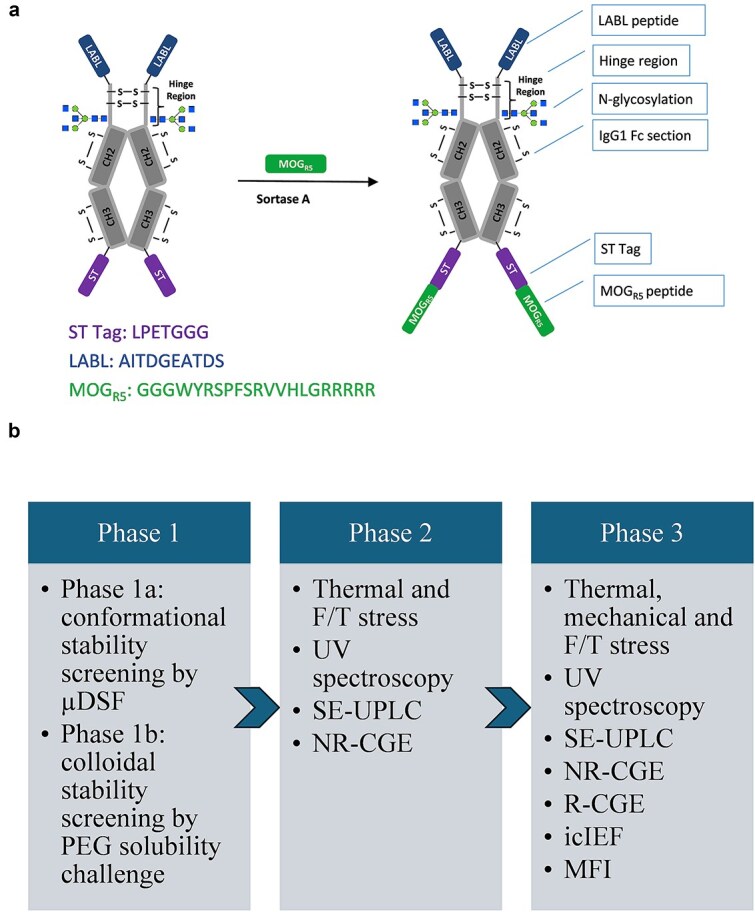
(a) Structural diagrams for sortase-mediate ligation of MOG_R5_ peptide with LABL-Fc-ST to form LABL-Fc-MOG_R5_ protein. Structural segments of the LABL-Fc-MOG_R5_ are indicated in the diagram. Amino acid sequences of ST tag, LABL peptide, and MOG_R5_ peptide are displayed as well. In actual IgG Fc structure, the N-linked glycosylation site occurs in the CH2 domain close to the hinge region. (b) A workflow of high-throughput protein formulation development. In Phase 1a, up to 96 formulations can be screened for conformational stability by microdifferential scanning fluorimetry (μDSF). In Phase 1b, up to 96 formulations can be screened for colloidal stability by polyethylene glycol precipitation assay (PEG solubility challenge). In Phase 2, up to 96 formulations can be screened in accelerated stability study up to 4 weeks at 25°C and freeze/thaw (F/T) stress, tested by ultraviolet (UV) spectroscopy, size-exclusion ultra-high performance liquid chromatography (SE-UPLC), and non-reduced-capillary gel electrophoresis (NR-CGE). Phase 3 is a formulation study as confirmation of previous observations including thermal, mechanical, and F/T stress. Testing for Phase 3 included UV spectroscopy, SE-UPLC, NR-CGE, reduced capillary gel electrophoresis (R-CGE), imaging capillary isoelectric focusing (icIEF), and microflow imaging (MFI).

Recently, an MOG-Fc-BPI called LABL-Fc-MOG_R5_ (Fig. 1a) was developed specifically for treating PPMS [11]. In the PPMS-induced EAE mouse model, LABL-Fc-MOG_R5_ demonstrated remarkable efficacy to reverse disease symptoms to a healthy level, highlighting its potential for addressing an unmet need in PPMS treatment [11]. Despite the excellent efficacy of the LABL-Fc-MOG_R5_ protein, it has poor physical stability in solution. For example, purified LABL-Fc-MOG_R5_ quickly formed a visible precipitate within an hour of purification in phosphate buffered saline. To maintain the stability of the LABL-Fc-MOG_R5_, various formulation conditions were investigated. A reasonably stable formulation was found in 100 mM sodium phosphate at pH 7.2 with 150 mM NaCl and 50 mM sucrose. This initial formulation maintained physical stability of the LABL-Fc-MOG_R5_ protein sufficiently to conduct *in vivo* evaluation in the EAE mouse model. Unfortunately, in the initial formulation, visible particles were observed within 24 h under refrigerated conditions [11]. The precipitation was exacerbated under mechanical stresses such as shaking and pipetting. Thus, the initial formulation of LABL-Fc-MOG_R5_ does not have sufficient shelf-life for long-term storage and cannot withstand commonly encountered stresses during drug administration. LABL-Fc-MOG_R5_ is a dimer with pI of 8.65 and molecular weight 58,770 Da under deglycosylated, non-reduced conditions and 29,391 Da under deglycosylated, reduced conditions. The N-terminal LABL peptide is negatively charged at pH 7.2 with two Asp residues and one Glu residue, while the C-terminal MOG_R5_ peptide is positively charged at pH 7.2 with seven Arg residues. In addition, the C-terminal MOG_R5_ peptide contains multiple residues with hydrophobic side chains such as Trp, Tyr, Phe, Leu, and Val. Thus, we postulate that the observed physical stability issues of LABL-Fc-MOG_R5_ are caused by different charge states at opposite ends of the protein leading to induced dipoles as well as hydrophobic interactions by the hydrophobic residues.

**Figure 2:**
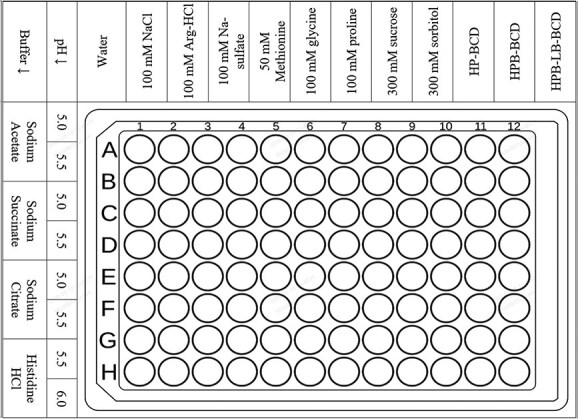
Plate design of biophysical screening for formulation development in Phase 1a and 1b. Four buffer systems were evaluated including sodium acetate, sodium succinate, sodium citrate, and histidine-HCl. Ranges of pHs from 5.0 to 6.0 depending on the buffering capacity of each buffer. Eleven different excipients were studied, including sodium chloride (NaCl), Arginine-HCl (Arg-HCl), sodium sulfate (Na-sulfate), methionine, glycine, proline, sucrose, sorbitol, HP-BCD, HPB-BCD, and HPB-LB-BCD.

In this study, the LABL-Fc-ST protein was expressed and purified following the procedure outlined in White *et al.*, 2017 [10]. A structural diagram for the sortase-mediated ligation of the MOG_R5_ peptide with LABL-Fc-ST to form the LABL-Fc-MOG_R5_ BPI is presented in Fig. 1a. The purified fusion protein underwent a three-phase formulation development process (Fig. 1b) to identify an optimal formulation suitable for long-term storage at refrigerated temperatures [12, 13]. High-throughput biophysical assays were employed to evaluate both conformational and colloidal stability in Phase 1. This stage examined a design space comprised of 96 distinct formulations, generated by combining twelve excipients with eight buffer and pH conditions (Fig. 2). Conformational stability reflects a protein’s resistance to unfolding [14, 15], whereas colloidal stability describes its tendency to interact or aggregate with other protein molecules [16, 17]. A PEG solubility challenge assay for this study was adapted from Xin *et al*., 2025 [13].

The Phase 2 study leveraged the conformational and colloidal stability determined in Phase 1 to further examine the protein stability via a design of experiments (DoE) approach to optimize the protein formulation. Six factors were investigated including two categorical and four numerical factors such as buffer type, buffer pH, tonicity modifier level, aggregation suppressor level, and polysorbate level. A definitive screening design (DSD) was employed to study the effect of the factors and the interaction between factors (Table 1). A DSD is highly efficient for experiments involving mixed-factor types. DSDs require a minimal number of runs—slightly more than twice the number of factors—and enable the estimation of main effects, interactions, and quadratic terms. Additionally, their capacity to detect nonlinear effects makes them particularly valuable for modeling complex systems [18]. Determination of protein aggregation and fragmentation was accomplished by size exclusion-ultra high-performance liquid chromatography (SE-UPLC) as well as non-reduced capillary electrophoresis (NR-CGE). In the final phase of the study, LABL-Fc-MOG_R5_ formulations were placed in pharmaceutical containers to undergo thermal, freeze/thaw, and mechanical stress. The stressed material was analyzed using SE-UPLC, R/NR-CGE, imaging capillary isoelectric focusing (icIEF), and microflow imaging (MFI) for subvisible particle (SVP) analyses.

**Table 1:** Definitive screening design for formulation optimization of LABL-Fc-MOG_R5_.

**No.**	**Block**	**Buffer pH**	**Sucrose (mM)**	**PS80 (%)**	**BCD (mM)**	**Glycine (mM)**
1	1	His-5.8	0	0.05	75	0
2	1	Ace-5.3	125	0.025	75	50
3	1	Ace-5.3	0	0	150	0
4	1	Ace-5.3	250	0.05	0	0
5	1	His-5.8	0	0	150	100
6	1	His-5.8	125	0.025	75	50
7	1	Ace-5.3	250	0	75	100
8	1	His-5.8	250	0.05	0	100
9	1	Ace-5.3	125	0.05	150	100
10	1	His-5.8	125	0	0	0
11	2	Ace-5.3	250	0.05	150	0
12	2	His-5.8	0	0	0	100
13	2	His-5.8	250	0.025	150	100
14	2	Ace-5.3	250	0	0	50
15	2	Ace-5.3	0	0.025	0	0
16	2	Ace-5.3	0	0.05	0	100
17	2	His-5.8	0	0.05	150	50
18	2	His-5.8	250	0	150	0
19	1	Current formulation control (100 mM sodium phosphate, pH 7.2, 150 mM NaCl, 50 mM sucrose)				
20	2					

## Materials and methods

### Materials

ACS reagent-grade chemicals, including sodium acetate, glacial acetic acid, sodium succinate, succinic acid, sodium citrate, citric acid, l-histidine, l-histidine hydrochloride, β-mercaptoethanol (BME), *N*-ethylmaleimide (NEM), hydrochloric acid, sodium hydroxide, sodium chloride, l-methionine, l-arginine, l-arginine hydrochloride, sodium sulfate, l-glycine, l-proline, sorbitol, and sucrose, were obtained from Sigma-Aldrich (St. Louis, MO, USA). Highly purified grades of polysorbate 20 and polysorbate 80 were sourced from NOF America (White Plains, NY, USA). Milli-Q ultrapure water was used for all experimental procedures. HP-βCD (KLEPTOSE® HP), HP-βCD low-substituted (HPB-BCD), and its lauroyl derivative (HPB-LB-BCD) were supplied by Roquette (Norristown, PA, USA).

### Production of LABL-Fc-MOG_R5_ BPI and purification using protein-a chromatography

LABL-Fc-MOG_R5_ protein was generated following the method described by White *et al*., 2017 [10]. LABL-Fc-ST, sortase enzyme, and the MOG peptide were prepared using previously established protocols at the University of Kansas. The identity of LABL-Fc-ST was confirmed by mass spectrometry (Supplementary Fig. S1a). The MOG_R5_ peptide was ligated to LABL-Fc-ST through a sortase-mediated transpeptidation reaction in a 125 ml shake flask at 37°C for 24 h. Protein-A affinity chromatography was performed using MabSelect SuRe™ LX resin (Cytiva, Marlborough, MA, USA) packed into a gravity-flow column (~1 ml resin volume). The column was pre-rinsed with three column volumes (CVs) of 50 mM Tris-acetate buffer (pH 7.4, 150 mM NaCl), sanitized for 15 min with 0.1 M NaOH (3 CVs), and re-equilibrated with 5 CVs of the Tris-acetate buffer. The load was adjusted to ~40 mg protein per ml of resin. Sequential washes included [1]: 3 CVs of 50 mM Tris-acetate (pH 7.4, 150 mM NaCl) [2], 5 CVs of 50 mM sodium acetate (pH 5.5, 1 M NaCl), and [3] 3 CVs of sodium acetate buffer at pH 5.5. Elution was carried out with 5 CVs of 50 mM sodium acetate (pH 3.6). The resin was then stripped with 1 M acetic acid (3 CVs), sanitized with 0.1 M NaOH (5 CVs), and stored in 20% ethanol at 5°C. Eluate fractions were immediately adjusted to pH ~5.5 using 1 M Tris (pH 9.0) at 4% (v/v) of the eluate volume. The final product was analyzed by mass spectrometry (Supplementary Figs S1 and S2).

### Formulation preparation, buffer exchange, and concentration of LABL-Fc-MOG_R5_

Buffer stock solutions (10× to 15×) were prepared at target pH values, while excipient stock solutions (5×) were adjusted to pH 6.0. All stock solutions were checked for pH and osmolality, then sterilized by 0.22 μm syringe filtration. Buffer plates (96- or 24-well) were assembled using an ASSIST PLUS automated pipetting system within a biosafety cabinet. LABL-Fc-MOGR5 was buffer exchanged into purified water using a μPulse automated buffer exchange system (Formulatrix, Bedford, MA, USA) at room temperature. Protein concentrations were quantified using a Lunatic plate-based spectrophotometer.

### Phase 1 study

#### Design of 96-well plate layout

The experimental layout (Fig. 2) screened sodium acetate, sodium succinate, and sodium citrate buffers at pH 5.0 or 5.5, and histidine-HCl buffers at pH 5.5 or 6.0. Each buffer was tested without excipients and in combination with eleven excipients, including salts (NaCl, arginine-HCl, sodium sulfate), amino acids (l-methionine, l-glycine, l-proline), sucrose, sorbitol, and β-cyclodextrins (KLEPTOSE® HP, HPB, HPB-LB).

#### Phase 1a: Melting temperature analyses by IF-DSF

Samples were heated from 25 to 95°C with increment of 0.25°C/min, and tryptophan fluorescence spectra (300–430 nm emission, 280 nm excitation) were recorded using a SUPR-DSF system (Applied Photophysics, UK). Data were analyzed via barycentric mean wavelength, with melting temperatures determined from first-derivative inflection points.

#### Phase 1b: Solubility assessment by PEG challenge

PEG-6000 was dissolved at 50% (w/v) in each buffer, then serially diluted to yield final PEG concentrations from 0% to 36% in 2% increments. Samples (1.0 mg/ml protein) were incubated for 3 h at 25 ± 3°C, centrifuged (1000 × *g*, 5 min), and turbidity (A350) was measured using a SpectraMax M5 reader. The highest PEG level yielding ≤0.2 AU at 350 nm was recorded as the precipitation threshold.

### Phase 2 and Phase 3 studies

#### Phase 2 study design

In Phase 2 study, a DSD was implemented to evaluate six variables: buffer type, pH, ionic strength modifier type and level, aggregation suppressor level, and polysorbate level. Two categorical and four numeric factors were included, allowing estimation of main effects, interactions, and quadratic terms while minimizing confounding. DSDs require a minimal number of runs—slightly more than twice the number of factors—and enable the estimation of main effects, interactions, and quadratic terms. By ensuring that main effects remain orthogonal to two-factor interactions, DSDs minimize confounding analysis and provide reliable estimates. Additionally, their capacity to detect nonlinear effects makes them particularly valuable for modeling complex systems [18].

#### Thermal, freeze–thaw, and mechanical stresses

During Phase 2, LABL-Fc-MOGR_5_ was buffer exchanged into Milli-Q water followed by filtration through 0.22 μm PES membrane prior to evaluation. Buffers were prepared at a concentration of 10× as a stock solution and a stock solution of excipients were prepared at a stock concentration of 3–10×. All formulations were combined in Nunc™ 96-well polypropylene storage plates (ThermoFisher, Waltham, MA) and heat-sealing foil (VITL, Ashland, VA) using HeatSealer (VITL, Ashland, VA) was utilized to seal them. In the formulation confirmation study in Phase 3, the buffer exchanged in all formulation was directly carried out into either the candidate formulation without polysorbate or the control formulation. Polysorbate was spiked in after buffer exchange. Then, 0.22 μm PES membrane was utilized to filter each formulation followed by hand-filling into 2R ready-to-use vials, stoppered, and crimped in vertical laminar flow hood. The thermal stress studies were accomplished using plates or vials in a calibrated incubator at 25°C/60%RH or 40°C/75%RH for up to 4 weeks. The samples were subjected to freeze–thaw stress; in this case, the plates were frozen at −80°C for 24 h, and then, they were thawed at 2–8 C for 24 h for a total of five cycles. Finally, the samples were subjected to mechanical stress; in this case, the vials were placed on a horizontal shaking plate and shook at 300 rpm for 72 h in the dark at room temperature of 25 ± 3°C.

#### Concentration analyses by ultraviolet (UV) spectroscopy

During Phase 2 and Phase 3 studies, protein concentrations were measured via Lunatic UV/Vis reader (low- or high-volume plates depending on concentration) or Cary-60 UV/Vis spectrophotometer with SoloVPE. An extinction coefficient of 1.32 ml·mg^−1^ cm^−1^ was used and only results with *R*^2^ ≥ 0.999 were accepted.

#### Size variant analyses by SE-UPLC

During Phase 2 and 3 studies, size variant analyses by SEC-UPLC was performed on an ACQUITY Premier Protein SEC column (Waters) at 25°C using 100 mM sodium phosphate (pH 7.2) with 200 mM arginine-HCl as the mobile phase (0.3 ml/min). Proteins were diluted to 1.0 mg/ml, centrifuged (3220 × *g*, 30 min, 10°C), and injected. Monomer, LMW, and HMW species were quantified using Empower 5.

#### Protein purity analyses by R/NR-CGE

During Phase 2 and 3 studies, the purity of the LABL-Fc-MOGR_5_ protein was measured by capillary electrophoresis sodium dodecyl sulfate under reducing and/or non-reducing conditions on a ProteinSimple® Maurice™ Plus Cartridge (ProteinSimple San Jose, CA). Samples were prepared in ProteinSimple sample buffer with NEM (non-reducing) or BME (reducing), spiked with 10 kDa standards, and run under programmed voltage steps. Specifically, the focusing was carried out by two steps [1]: 4.6 kV for 20 s [2]; 5.75 kV for 35 min (non-reduced) or 30 min (reduced), with autosampler temperature maintained at 15°C. Data were processed with Compass for iCE under UV mode.

#### Charge variant analyses by icIEF

Charge heterogeneity was evaluated using imaging capillary isoelectric focusing (icIEF) on a ProteinSimple® Maurice™ system (San Jose, CA, USA). Both UV absorbance at 280 nm and native fluorescence spectra were recorded for each sample. For analysis, 20 μl of the reference standard or test sample (diluted to 1.0 mg/ml) was combined with 80 μl of a master mix containing: 1.0 μl of low pI marker (pI 7.05), 1.0 μl of high pI marker (pI 10.17), 2.0 μl Pharmalyte 3–10, 2.0 μl Pharmalyte 8–10.5, 35 μl of 1% methylcellulose (MC), 25 μl of 8 M urea, 3.0 μl of 200 mM arginine-HCl, 4.0 μl of 500 mM arginine-HCl, and 7 μl of ultrapure water. This preparation yielded a final protein concentration of 0.2 mg/ml in the loading mixture. Samples were introduced into a Maurice™ cIEF cartridge, and focusing was performed in two voltage steps: 1.5 kV for 1 min, followed by 3 kV for 9 min, with the autosampler tray maintained at 10°C. Detection was carried out via both UV280 and native fluorescence modes. Fluorescence data captured at 10 seconds were processed using either Empower Software or Compass for iCE.

#### SVP measurement

SVP size distribution, count, and morphology were determined using a ProteinSimple® Micro-Flow Imaging (MFI) instrument (San Jose, CA, USA). Samples were loaded into a 96-well plate for automated imaging and analysis. All manipulations were conducted inside a biosafety cabinet to minimize contamination. Between measurements, the flow cell was cleaned with 2% Liquinox detergent, rinsed thoroughly with particle-free water, and baseline performance was verified. For each measurement, 1.0 ml of sample was dispensed, of which 0.5236 ml was analyzed. Results were reported as particle counts per container for the following size categories: ≥2, ≥5, ≥10, and ≥25 μm.

#### Analysis of the intact mass of reduced and deglycosylated protein

The molecular weight of the LABL-Fc-MOG_R5_ was determined by the intact mass spectrometry of reduced-deglycosylated protein. For each sample, 40 μg of protein samples at 1.0 mg/ml were reduced with 25 mM dithiothreitol in 1× Rapid PNGase F Buffer (New England BioLabs, Ipswich, MA) at 75°C for 8 min. After reduction, 2.0 μl of Rapid PNGase F (New England BioLabs, Ipswich, MA) was added to each 40 μg of protein and incubated at 50°C for 15 min. Following the deglycosylation, the reaction mixture was diluted to 0.4 mg/ml protein concentration with Milli-Q water. Intact-MS analyses were performed with Waters ACQUITY UPLC Protein BEH C4 Column, 300 Å, 1.7 μm, 2.1 mm × 50 mm with Waters ACQUITY H-Class UPLC with binary pump, PDA and RDa detector. Mobile phase A was LC–MS grade water with 0.1% formic acid. Mobile phase B was LC–MS grade Acetonitrile with 0.1% formic acid. For each analysis, 10 μl of sample were injected and the analyses gradient is listed in Table 2.

**Table 2:** Gradient for intact mass analyses by waters ACQUITY UPLC.

**Time (min)**	**%A**	**%B**	**Flow rate (ml/min)**
***0.0***	80.0	20.0	0.4 ml/min
***1.0***	80.0	20.0	
***8.0***	50.0	50.0	
***11.0***	10.0	90.0	
***13.0***	10.0	90.0	
***13.1***	80.0	20.0	
***15.0***	80.0	20.0	

#### Statistical analyses

Data analysis was conducted using JMP version 17.0.0 (JMP Statistical Discovery LLC, Cary, NC) and GraphPad Prism (GraphPad Software, Boston, MA). Multivariate analyses were carried out using a row-wise approach, with buffer type, pH, buffer–excipient interactions, and pH–excipient interactions included as factors in the model fitting. Effect screening was applied to develop the profiling tool. Unpaired parametric t-tests and graphical outputs were generated with both GraphPad Prism and the JMP graphing functions.

## Results

### Phase 1: Biophysical screening for protein conformational and colloidal stability

In this phase of the study, LABL-Fc-ST and LABL-Fc-MOG_R5_ were measured for conformational and colloidal stability under a panel of formulations (Fig. 2). LABL-Fc-ST was studied alongside of LABL-Fc-MOG_R5_ because the precipitation issues observed for LABL-Fc-MOG_R5_ in the control formulation were not observed for LABL-Fc-ST during any of the handling steps including repeated freeze/thaw and agitation. The primary sequences of LABL-Fc-ST and LABL-Fc-MOG_R5_ differ only in the addition of the MOG_R5_ peptide to the C-terminus by sortase ligation. By evaluating both structures together in the biophysical screening, we hope to isolate any conformational or colloidal stability issues upon the addition of the MOG_R5_ peptide to the C-terminus of LABL-Fc-ST, thus changing the overall biophysical properties of the molecule.

#### Phase 1a: Protein conformational stability

**Figure 3:**
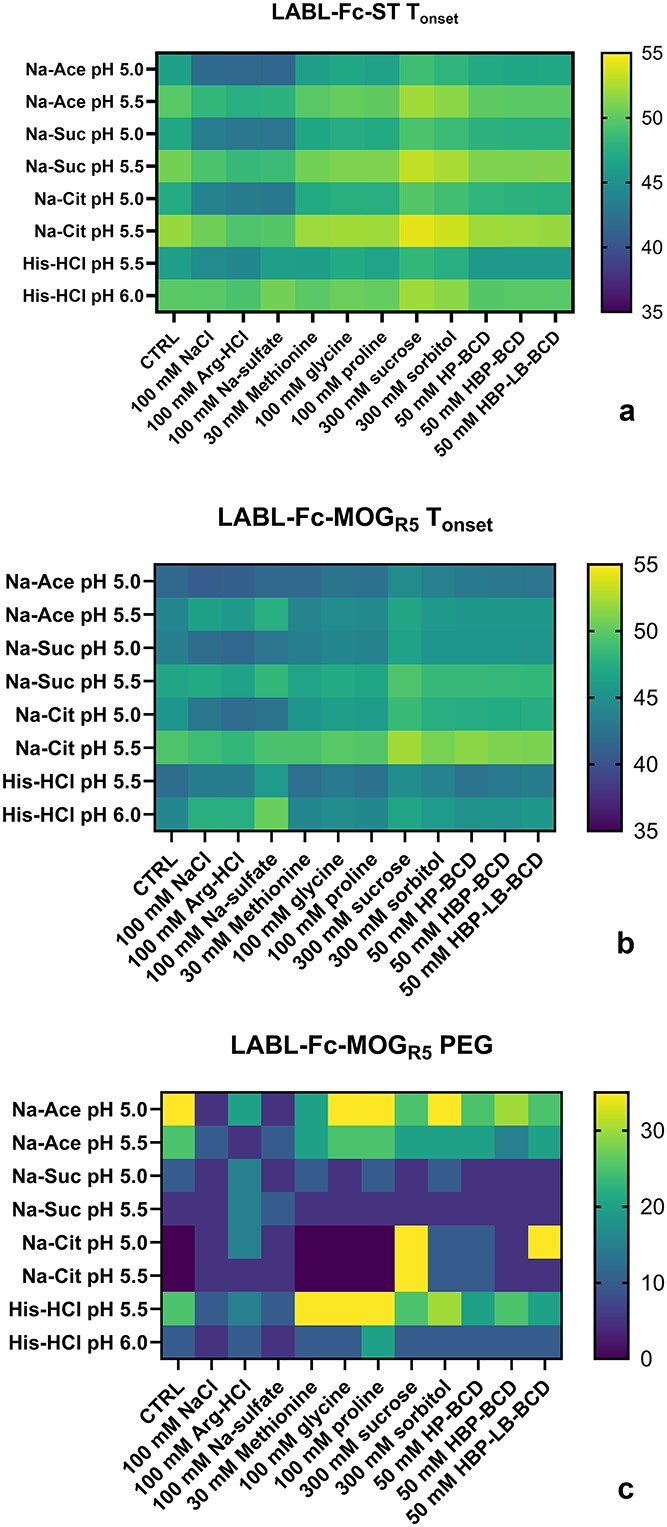
Conformational stability by *T*_onset_ analysis for (a) LABL-Fc-ST and (b) LABL-Fc-MOG_R5_ and colloidal stability for LABL-Fc-MOG_R5_ in 96 different formulations. The data are presented in heat map format with dark purple representing lower *T*_onset_ at 35°C and yellow representing higher *T*_onset_ of 55°C. The average *T*_onset_ value for LABL-Fc-MOG_R5_ is 45.6 ± 2.7°C and the average *T*_onset_ value for LABL-Fc-ST is 48.5 ± 2.8°C. (c) PEG data are presented as %PEG at which visible turbidity was observed in the sample.

Protein conformational stability—the ability to maintain its 3D structure—is crucial for long-term stability, ensuring structural and functional integrity over time. Conformational stability of proteins is affected by formulation, and inherent poor conformational stability could lead to denaturation, aggregation, and loss of function. In this study, we used intrinsic fluorescence-differential scanning fluorimetry (IF-DSF) to measure the onset of protein denaturation; the *T*_onset_ results for LABL-Fc-ST and LABL-Fc-MOG_R5_ showed similar trends between the two molecules in different buffers/pH combinations (Fig. 3a and b and Supplementary Fig. S3). Specifically, in each of the four buffers, increasing pH by 0.5 unit resulted in the increase of *T*_onset_ in both LABL-Fc-ST and LABL-Fc-MOG_R5_. Among the four buffer groups, the *T*_onset_ ranking is as follows: Na-acetate ≈ histidine-HCl < Na-succinate < Na-citrate and this trend was more pronounced in LABL-Fc-MOG_R5_ compared to that of LABL-Fc-ST (Supplementary Fig. S3).

LABL-Fc-ST and LABL-Fc-MOG_R5_ showed different responses to excipient groups. For LABL-Fc-ST, compared to the water control, both sucrose and sorbitol increased the *T*_onset_, while NaCl, arginine-HCl, and Na-sulfate lowered the *T*_onset_. l-Methionine, l-glycine, l-proline, and the three types of HP-βCD did not show differences in *T*_onset_ compared to control (Fig. 3a). For LABL-Fc-MOG_R5_, *T*_onset_ was increased by sucrose, sorbitol, Na-sulfate, and the three types of HP-βCD; in contrast, NaCl, arginine-HCl, l-methionine, l-glycine, and l-proline did not show differences compared to the control (Fig. 3b).

#### Phase 1b: Protein colloidal stability

Protein colloidal stability refers to the ability of proteins to remain dispersed without forming aggregates in solution. Aggregation reduces solubility, alters bioactivity, and complicates therapeutic protein formulation. In this study, a PEG challenge assay was adapted from Xin *et al.*, 2025 [13]. The results from PEG solubility challenge for LABL-Fc-MOG_R5_ in 96 different formulations are presented in Fig. 3c. Although this assay showed greater variability than the *T*_onset_ assay, it was apparent that within the same buffer group lower pH is favorable, i.e. a lower %PEG is required to induce turbidity at a higher pH than at lower pH. In addition, divalent buffers were generally unfavorable, requiring a lower %PEG to induce precipitation of LABL-Fc-MOG_R5_ compared with monovalent buffers.

Considering the results of Phase 1a and 1b, Na-acetate and histidine-HCl with low ionic strength buffers were chosen to maximize colloidal stability. For both buffer systems, the mid-point pH at 5.3 or 5.8 from Phase 1 was chosen for further evaluation. Sucrose and glycine were included for conformational stability of both LABL-Fc-ST and LABL-Fc-MOG_R5_. Furthermore, hydroxypropyl-β-cyclodextrin (HP-βCD) was included for maintaining conformational stability of LABL-Fc-MOG_R5_. Of the three types of beta-CD, HP-βCD with lauroyl modification (HBP-LB-BCD) was selected to move forward based on literature suggestions [19]. HBP-LB-BCD, by combining hyperbranched architecture and lipid-bearing moieties, is predicted to provide multivalent, steric shielding, offering maximal protection against aggregation, including in high-stress (heat/light/agitation) conditions [20]. Polysorbate 80 (PS80) was included to protect against interfacial stress. The chosen parameters were combined in a definitive screening DoE design comprised of the conditions in Table 1. The corresponding statistical design evaluation is found in Supplemental Fig. S4.

#### Phase 2: DoE-based formulation optimization study

**Figure 4:**
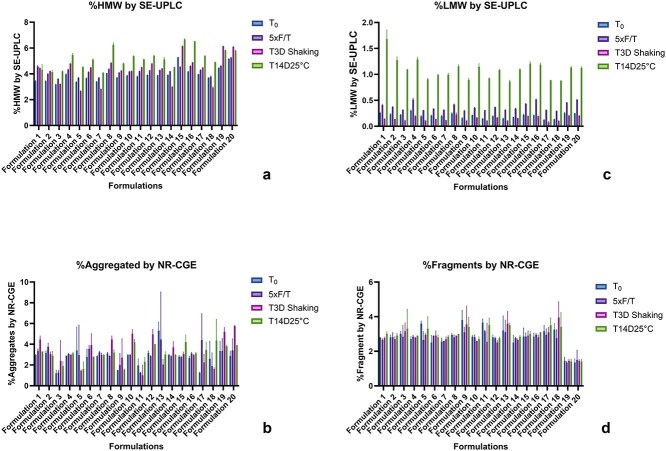
Stability indicating assay results for DSD formulation optimization design. Protein aggregation is represented by (a) %HMW by SE-UPLC and (b) %aggregation by NR-CGE. Protein fragmentation is represented by (c) %LMW by SE-UPLC and (d) %fragmentation by NR-CGE. For SEC assay, samples are tested with triplicate preparations, and the assay variation is represented by the error bars from standard deviations calculated based on *n* = 3. For NR-CGE assay, samples are tested with duplicate preparations, and the assay variation is represented by the error bars from standard deviations calculated based on *n* = 2. T0 represents the initial condition. 5×F/T designation represents measurement after five cycles of freeze/thaw. T3D shaking represents measurements after 72 ± 6 h of shaking at 300 rpm in dark at room temperature (~25 ± 3°C). T14D25°C represents measurements after 14 days storage at 25°C/60% relative humidity (RH).

To optimize the formulation composition, LABL-Fc-MOG_R5_ at 2.5 mg/ml was subjected to accelerated stability conditions to stress the protein with conditions tested consisting of shaking at 300 rpm for 3 days at 25°C, five cycles of freeze/thaw, or 2 weeks at 25°C/60%RH. After accelerated stress, proteins often show elevated aggregation levels that can be used to infer a protein’s tendency to form aggregates during long-term storage or manufacturing processes. Aggregate formation does not only decrease the potency of the protein, but it may cause undesired immune reactions. In the Phase 2 study, we measured protein aggregation of high-molecular weight species (%HMW) using SE-UPLC (Fig. 4a) and aggregate size by NR-CGE (Fig. 4b). Formulations 3, 5, 7, 14, and 18 showed overall lower levels of %HMW compared to the control formulations (Fig. 4a and b and Supplementary Fig. S5a).

Accelerated conditions may also lead to protein fragmentation, particularly under elevated temperature stress. Fragmented protein is often associated with reduced potency and loss of function. In the Phase 2 study, we assessed low-molecular weight species (%LMW) by SE-UPLC (Fig. 4c) and %fragmentation by NR-CGE (Fig. 4d) to get an overall measure of LABL-Fc-MOG_R5_ fragmentation. %LMW determined by SEC did not show significant differences among the formulations due to large assay variabilities (Fig. 4c and Supplementary Fig. S5b). Using NR-CGE, formulation 3, 5, 9, 11, 17, 18 showed lower levels of aggregates as measured by NR-CGE compared to the control (Fig. 4d and Supplementary Fig. S5c). However, formulations 3, 5, 6, 7, 9, 11, 13, 17, and 18 showed lower %LMW compared to the control, when only considering the %LMW measured at T2W25°C (Fig. 4d). By NR-CGE, all the experimental formulations exhibited higher fragmentation levels compared to the control formulation (Supplementary Fig. S5d).

**Figure 5:**
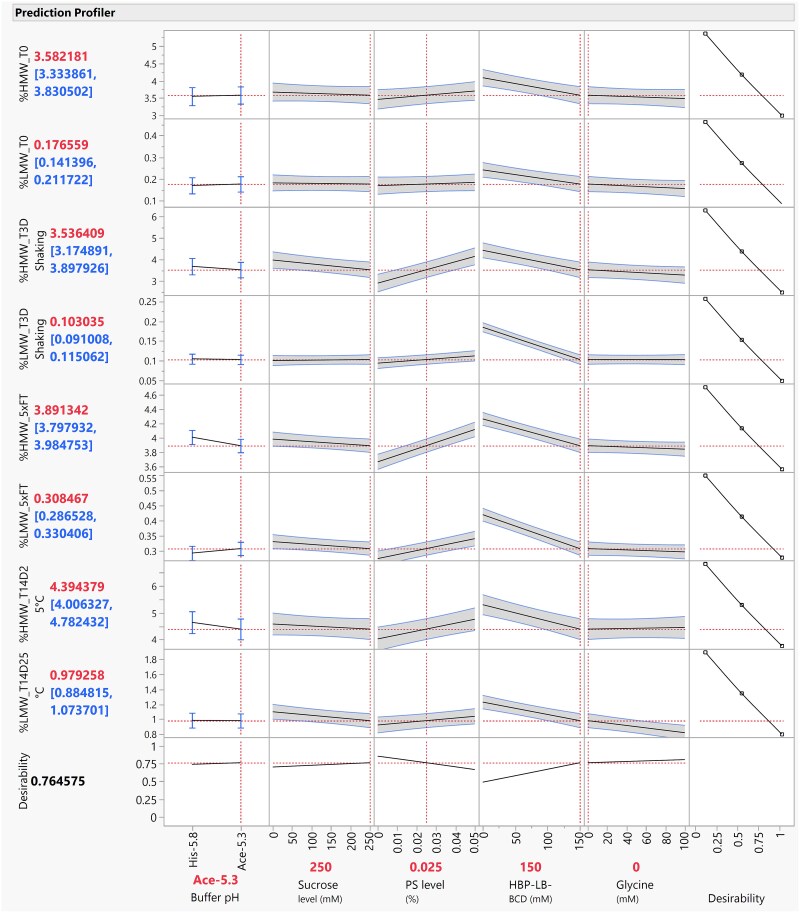
Stability indicating assay results from DSD-based formulation optimization study. Desirability function was set at minimizing %HMW and %LMW at different timepoints. The final presented condition represents the most favorable condition statistically. %HMW and %LMW represent percent high-molecular weight species and low molecular weight species, respectively, that were measured by SE-UPLC. T0 represents the initial condition. 5×F/T represents measurement after five cycles of freeze/thaw. T3D shaking represents measurements after 72 ± 6 h of shaking at 300 rpm in dark at room temperature (~25 ± 3°C). T14D25°C represents measurements after 14 days storage at 25°C/60% RH.

A closer look at the statistical analysis (Fig. 5) shows that Na-acetate buffer pH 5.3 had lower %HMW and higher %LMW as determined by SE-UPLC compared to histidine-HCl buffer at pH 5.8 after 5× F/T and T2W25°C stress. Sucrose appeared to protect against LMW fragment formation, but the trend was not statistically significant. Addition of HBP-LB-BCD showed benefits in suppressing both the aggregation and fragmentation. Interestingly, increasing PS80 levels were associated with increased aggregation and fragmentation.

Leveraging the statistical analysis results, a formulation consisting of acetate buffer at pH 5.3, 125 mM sucrose, and 0 mM glycine were chosen for further evaluation. Notably, statistical analyses identified that 250 mM sucrose was an optimal condition. To maintain osmolality close to an isotonic level, the concentration of sucrose was reduced to 125 mM; furthermore, only slight differences in protein aggregation or fragmentation were detected between formulations containing 125 and 250 mM sucrose. A maximum level of HBP-LB-BCD at 150 mM and 0.025% w/v of PS80 was added to the formulation. The new formulation was designed at a 10 mg/ml concentration of LABL-Fc-MOG_R5_ at pH 5.3 in 10 mM sodium acetate, 125 mM sucrose, 150 mM HBP-LB-BCD, and 0.025% PS80. This new formulation was compared against the control formulation of 100 mM sodium phosphate, pH 7.2, 150 mM NaCl, and 50 mM sucrose. The new formulation at pH 5.3 with a theoretical osmolality of 300 mOsm/kg is suitable for drug delivery by intravenous, intramuscular, intraperitoneal, and subcutaneous injections.

#### Phase 3: Formulation confirmation study

**Figure 6:**
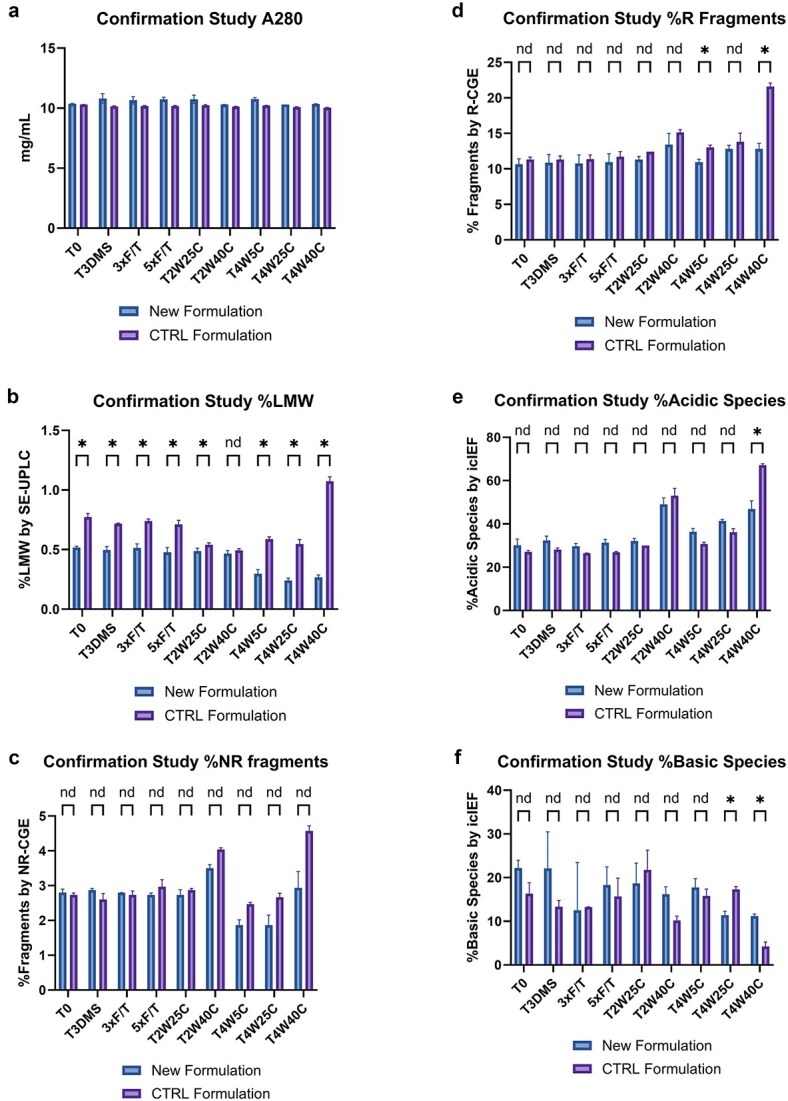
Fragments formation, charge variants, and concentration results for LABL-Fc-MOG_R5_ in new (F1) and control (F2) formulation by multiple assays. (a) Concentration was measured by A280 and presented as mg/ml. Fragmentation was measured by (b) %LMW using SE-UPLC, (c) %fragmentation by NR-CGE, and (d) %fragmentation by R-CGE. Charge variants are measured by (e) %acidic and (f) %basic using icIEF. Error bars represent single analyses of triplicate samples. T0 represents the initial condition. 3×F/T and 5×F/T represent measurement after three or five cycles of freeze/thaw. T3DMS represents measurements after 72 ± 6 h of shaking at 300 rpm in dark at room temperature (~25 ± 3°C). T2W25C and T4W25C represent measurements after 14 or 28 days storage at 25°C/60% RH, respectively. T2W40C and T4W40C represent measurements after 14 or 28 days storage at 40°C/75% RH, respectively. T4W5C represents measurements after 28 days storage at 2–8°C. Statistical significance was assessed using multiple unpaired *t*-tests comparing the control and new formulation across the test panel. “nd” indicates no statistically significant difference, while “^*^” indicates a statistically significant difference. The false discovery rate (FDR) was controlled at 1%. Multiple unpaired two-tailed t-tests with Benjamini–Hochberg FDR correction (q = 1%). ^*^ indicates FDR-adjusted *P* < 0.01.

This study was designed to confirm that the new formulation for LABL-Fc-MOG_R5_ would outperform the initial control formulation upon increasing concentrations from 2 to 10 mg/ml in different storage containers (i.e. polypropylene plate and borosilica glass vials). The results indicate that there is no protein concentration change in low or high concentrations at all time points. This indicated that higher concentration and good container closure produced formulation consistency. Therefore, concentration differences were not considered for evaluating the degradation rates (Fig. 6a).

Protein fragmentation is monitored during formulation development since increased levels of fragmentation can lead to reduced potency, loss of function, and undesired immunogenicity. Fragmentation was measured by several orthogonal assays including %fragments by NR-CGE, %LMW by SE-UPLC, and %fragments by R-CGE. Results by SE-UPLC (Fig. 6b), NR-CGE (Fig. 6c), and R-CGE (Fig. 6d) showed lower levels of fragments in the new formulation compared to the control formulation. By SE-UPLC, %LMW was found to be significantly lower in the new formulation in all timepoints, except for the T2W40°C timepoint (Fig. 6b  *P* < FDR of 1%). By NR-CGE, T2W40°C, T4W5°C, T4W25°C, and T4W40°C stresses appear to show higher levels of fragmentation, but the difference was not statistically significant considering assay variations (Fig. 6c, *P* ≥ FDR of 1%). Finally, analyzing the fragments under reduced conditions showed significantly higher levels of fragments in the control formulation compared to the new experimental formulation in T4W5°C and T4W40°C (Fig. 6d, *P* < FDR of 1%). Of note, compared to T0, fragmentation levels in the new formulation remained unchanged for all the stresses in all assays, while the fragmentation levels in the control formulation increased significantly under 40 °C/75%RH stress. Overall, higher levels of fragments were found in the control formulation compared to the new formulation.

In addition to protein fragmentation, many post-translational modifications (PTMs) such as Asn deamidation, Lys glycation, N-glycosylation, and Asp isomerization can be observed as charge variants. Tracking changes in charge variants in protein drugs during stability studies help drug designers to understand the chemical changes to the protein in different formulations. Charge variant analyses were carried out using an icIEF assay to indicate the formation of acidic species. Compared to T0, charge variants increased significantly under 40°C/75%RH stress. At T4W40°C, significantly higher levels of acidic species were found in the control formulation compared to the new formulation (Fig. 6e, *P* < FDR of 1%). After stress, the increase in %acidic species was accompanied by a decrease in basic species (Fig. 6f). Compared to T0, the %basic species decreased for all stress conditions in the new formulation. For the control formulation, the %basic species increased over T0 in T2W25C and T4W25C conditions (Fig. 6f).

**Figure 7:**
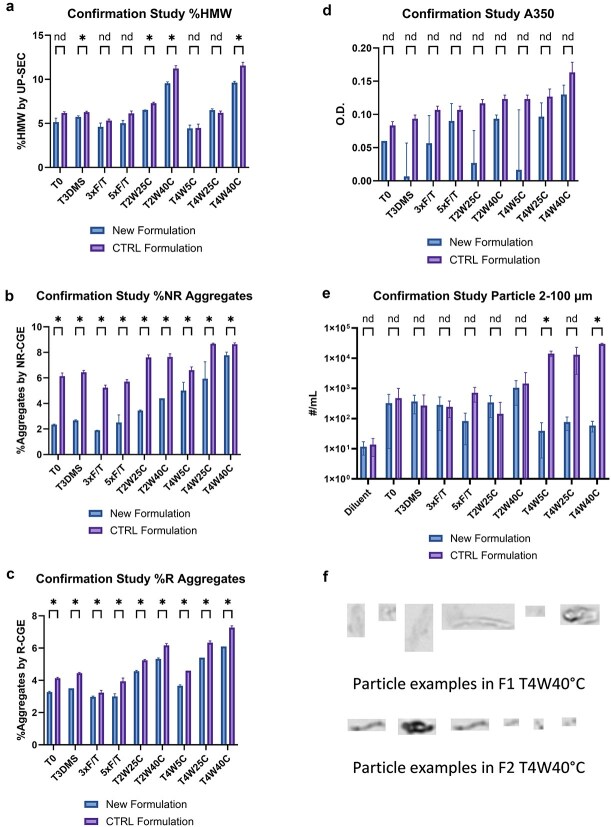
The formation of aggregates from LABL-Fc-MOG_R5_ in new (F1) and control (F2) formulations evaluated by multiple assays. Soluble aggregates were measured by SE-UPLC and presented as (a) %HMW; (b) aggregates in non-reduced; and (c) reduced CGE. (d) Solution turbidity was presented as A350. (e) Subvisible particles between 2 and 100 μm. (f) Representative of particle images. SVP counts were presented on Log10 scale and other attributes were plotted in linear scales. Error bars represent single analyses of triplicate samples. T0 represents the initial condition. 3×F/T and 5×F/T represent measurement after three or five cycles of freeze/thaw. T3DMS represents measurements after 72 ± 6 h of shaking at 300 rpm in dark at room temperature (~25 ± 3°C). T2W25C and T4W25C represent measurements after 14 or 28 days storage at 25°C/60% RH, respectively. T2W40C and T4W40C represent measurements after 14 or 28 days storage at 40°C/75% RH, respectively. T4W5C represents measurements after 28 days storage at 2–8°C. Statistical significance was assessed using multiple unpaired *t*-tests comparing the control and new formulation across the test panel. “nd” indicates no statistically significant difference, while “^*^” indicates a statistically significant difference. The false discovery rate (FDR) was controlled at 1%. Multiple unpaired two-tailed t-tests with Benjamini–Hochberg FDR correction (q = 1%). ^*^ indicates FDR-adjusted *P* < 0.01.

Another key factor in long-term stability is protein aggregation, as elevated levels of soluble aggregates or SVPs may reduce biological activity and increase immunogenicity. Aggregation was measured by several orthogonal assays including %HMW by SE-UPLC (Fig. 7a), %aggregates by NR-CGE (Fig. 7b), %aggregates by R-CGE (Fig. 7c), turbidity by UVA350 (Fig. 7d), and SVP analyzed by MFI (Fig. 7e). The overall results indicate that the new formulation was less susceptible to aggregate formation under stress conditions compared to the control formulation. Compared to T0, for both new and control formulations, various measures of aggregation were increased in the accelerated stability at 25°C/60%RH and 40°C/75%RH, including soluble aggregates by %HMW (Fig. 7a), aggregates in NR-CGE (Fig. 7b), aggregates by R-CGE (Fig. 7c), solution turbidity (Fig. 7d), and SVPs (Fig. 7e). Using SE-UPLC, higher levels of %HMW were found in the control formulation compared to the new formulation after mechanical stress, 2-week 25°C/60%RH stress, 2-week 40°C/75%RH stress, and 4-week 40°C/75%RH stress (Fig. 7a, *P* < FDR of 1%). Next, the NR-CGE assay was used to analyze the aggregates that were covalently linked. Higher levels of %aggregates were found in the control formulation compared to the new formulation in all timepoints including T0. This result indicates the instability of LABL-Fc-MOG_R5_ with its tendency to form covalent-linked aggregates in the control formulation (Fig. 7b, *P* < FDR of 1%). The R-CGE assay was used to analyze covalently linked aggregates that are not related to disulfide bonds. Higher levels of %aggregates were found in the control formulation compared to the new formulation at all timepoints including T0. This suggests a greater tendency of LABL-Fc-MOG_R5_ to aggregate in the control formulation (Fig. 7c, *P* < FDR of 1%). Using UV-A350, the solution turbidity was higher in the control formulation compared to the new formulation at all time points; due to large assay variation, there was no observed statistical significance (Fig. 7d). In addition, no visible particles were observed in the control or the new formulations in the Phase 3 study. However, a significant number of visible particles were observed when the control formulation samples were diluted for analytical assays. These particles were removed by centrifugation prior to sample analysis.

Finally, higher levels of SVPs were found in the control formulation compared to the new experimental formulation, and the data comparisons were statistically significant for the T4W5°C and T4W40°C conditions (Fig. 7e, *P* < FDR of 1%). At T4W25°C, the control formulation exhibited higher SVP counts compared with the new formulation. SVPs were found in both the new and the control formulation and they appeared to be white colored and translucent amorphous particles, which are characteristic of proteinaceous particles (Fig. 7f). Overall, the new formulation of LABL-Fc-MOG_R5_ was considered more effective at curbing aggregation and particle formation than the control formulation. Therefore, considering the multiple orthogonal results from different types of stresses of LABL-Fc-MOG_R5_, the final formulation was chosen to be 10 mg/ml of LABL-Fc-MOG_R5_ at pH 5.3 in 10 mM sodium acetate, 125 mM sucrose, 150 mM HBP-LB-BCD, and 0.025% PS80.

## Discussion

The formulation development study described here for the LABL-Fc-MOG_R5_ protein effectively addresses its physical instabilities, such as its tendency to aggregate under conventional conditions. Initial work with LABL-Fc-MOG_R5_ revealed a rapid precipitation of the purified protein; thus, it was necessary to develop a robust formulation. The selected formulation is intended to maintain protein integrity against various stresses encountered during processing, handling, and storage. It provides stability to the LABL-Fc-MOG_R5_ molecule during repeated freeze–thaw cycles, mechanical agitation, and 4 weeks of storage at 5°C, without a notable rise in protein aggregation (Fig. 7a), particle formation (Fig. 7e), fragmentation (Fig. 6b) or charge variants (Fig. 6e and f). For the 25°C/60%RH stress, the final formulation also protected LABL-Fc-MOGR_5_ from fragmentation and formation of charge variants (Fig. 6), while a moderate increase in particles and aggregates was observed (Fig. 7).

When comparing the candidate formulations with the initial control formulation, the most revealing condition was at 40°C and 75% RH (40°C/75%RH). Under this stress condition for 4 weeks, the control formulation (150 mM NaCl, 100 mM sodium phosphate, and 50 mM sucrose at pH 7.2) showed significant increase in fragmentation (Fig. 6b), acidic species formation (Fig. 6d), aggregation (Fig. 7a), and particle formation (Fig. 7e). In contrast, the final formulation showed no increase fragmentation (Fig. 6b–d) or acidic species formation (Fig. 6e). The increase in soluble aggregates as detected by SE-UPLC, NR-CGE, or R-CGE was significantly lower in the final formulation compared to the control formulation (Fig. 7a–c). Finally, there were several orders of magnitude less SVPs in the final formulation compared to the control formulation with stress condition at 40°C/75%RH (Fig. 7e).

The optimization of the LABL-Fc-MOG_R5_ formulation was initiated with the design of the Phase 1 formulation study (Fig. 2). Chemical degradation of the Fc subunit, LABL peptide (AITDGEATDSG), and MOG peptide (GWYRSPSRVVHL) were considered when choosing the pH range for the study. For example, the Asn (N) residues in the “PENNY” sequence of the IgG1 Fc CH2 region are prone to a deamidation reaction at pH 6.0 or above [21]. In addition, multiple aspartic acid (D) residues in the LABL peptide were susceptible to fragmentation at pH below 4.0 [22]. Finally, the hinge region of IgG1 Fc is also prone to fragmentation at low pH, and this phenomenon is exacerbated by addition of certain anions [12, 23]. Taking into consideration these chemical degradation reactions, the formulation study of LABL-Fc-MOG_R5_ was carried out at a pH range between 5.0 and 6.0 to maximize chemical stability. Different buffer systems were included to cover the similar pH ranges to study the specific effect of monovalent (sodium acetate and histidine-HCl) compared to bivalent buffers (sodium succinate and sodium citrate) [12].

The effects of different classes of excipients were evaluated, including salts, amino acids, amino acid salts, sugar, polyol, and β-cyclodextrins. NaCl is a common salt used to adjust the ionic strength of formulations and to leverage its “salting-in” effect to maintain solubility [24]. Sodium sulfate is often used as an alternative anion to the chloride ion because of its counter ion interactions with amino acids [12, 25]. Arginine-HCl has been added to formulations because it has been shown to prevent protein–protein interactions and aggregation [26–28]. To reduce oxidative stress-induced aggregation, methionine has been added into formulations as an excipient [29]. Both glycine and proline have been shown to reduce surface hydrophobic–hydrophobic interactions during protein aggregation [26].

In the Phase 1a study, the observed higher *T*_onset_ in divalent buffers compared to monovalent buffers could be due to the ionic effect in stabilizing salt-bridges in LABL-Fc-ST and LABL-Fc-MOG_R5_. The increased ionic strength in the divalent buffers may shield surface-exposed amino acids and allow the protein to maintain a compact and stable conformation [30, 31]. Compared to LABL-Fc-ST, LABL-Fc-MOG_R5_ has five additional surface-exposed arginine residues with positive charges that can be shielded in higher ionic strength buffers. These may be the reasons for the more pronounced differences in *T*_onset_ among the different buffer groups. Overall, conjugating the MOG_R5_ peptide onto LABL-Fc-ST lowered the *T*_onset_ of LABL-Fc-MOG_R5_ (45.6 ± 2.7°C) compared to LABL-Fc-ST (48.5 ± 2.8°C) (Fig. 3).

In the Phase 1b study, LABL-Fc-MOG_R5_ showed improved colloidal stability in low ionic strength buffers such as histidine-HCl and Na-acetate. We proposed that this was due to enhanced electrostatic repulsion between similarly charged surface residues. It was hypothesized that the colloidal stability of LABL-Fc-MOG_R5_ was governed by electrostatic repulsion, and the higher ionic strength environment shielded the surface charges to reduce electrostatic repulsion. At lower salt concentrations, fewer ions are available to shield surface charges, leading to stronger repulsive forces that prevent protein–protein interactions and aggregation [32, 33]. Finally, different excipients did not show dramatically different %PEG values.

We hypothesized that the aggregation problem of LABL-Fc-MOGR_5_ was partially due to the hydrophobic nature of the MOG_R5_ peptide that was conjugated to the parent LABL-Fc-ST protein. HP-βCD has been shown to be a potent aggregation suppressor [20, 34]. Thus, the hydrophobicity problem introduced by the MOG_R5_ peptide may be mitigated by complexation of its aromatic groups with HP-βCD to prevent protein aggregation and fragmentation. In this study, HP-βCD increased *T*_onset_ in LABL-Fc-MOGR_5_ but not in LABL-Fc-ST, suggesting that the hydrophobicity of MOGR_5_ peptide contributed to the aggregation of the LABL-Fc-MOGR_5_ protein. Presumably, the hydrophobic cavity of HP-βCD formed an inclusion complex with hydrophobic residues (i.e. Trp, Tyr, Val, Leu, Pro) on the MOG_R5_ fragment of the LABL-Fc-MOGR_5_ protein [35]. As a result, the complex could prevent hydrophobic-hydrophobic interactions between partially unfolded proteins, and prevent nucleation of the aggregation process.

In the Phase 2 study, the observed association between increasing sucrose concentration and lower fragmentation was consistent with literature reports; thus, the protein conformational stability was due to the sucrose exclusion mechanism to enhance water packing on the protein surface [36, 37]. The lower levels of %HMW detected by SE-UPLC in the Na-acetate buffer at pH 5.3 versus the histidine-HCl buffer at pH 5.8 were due to a higher positive charge on LABL-Fc-MOG_R5_ at pH 5.3 compared to that of at pH 5.8 (given the pI of the molecule at 8.65). Thus, this improved the protein colloidal stability, and it is correlated with findings by Xin *et al*., 2024 and 2025 [12, 13]. Sucrose is one of the most widely used stabilizers for protein therapeutics [24, 37]. Sucrose stabilizes native conformation of proteins through a preferential exclusion mechanism to enhance surface hydration of proteins and reduce protein mobility [36]. Sucrose can also facilitate the formation of an amorphous glassy matrix upon drying to reduce protein molecular mobility [36, 37]. Sugar alcohols (i.e. sorbitol and mannitol) have similarly been widely used as protein stabilizers via preferential exclusion and protein hydration mechanisms [38].

In the Phase 2 study, we also observed an association between increasing PS80 levels with increased aggregation and fragmentation (Fig. 5). It is generally believed that PS80 protects proteins against interfacial stress and reduces particles in formulation [20, 39, 40]. Although the association between protein fragmentation and polysorbate addition is not commonly reported, there are several proposed explanations for the effects of increased PS80 concentrations on aggregation and fragmentation. First, one potential reason is that different basic protein structures (i.e. primary, secondary, tertiary) respond differently to increased PS80 in their aggregation and fragmentation profiles. Second, the effects of PS80 on protein stability can be influenced by physicochemical properties (i.e. charge and hydrophobicity). Third, the quality of PS80 can influence the protein stability in formulation; PS80 has been shown to degrade over time, and its degradation products can stimulate protein physical instability. Degraded PS80 also has lower capacity to provide interfacial stress protection compared to undegraded PS80 [41]. Finally, degraded PS80 produces free fatty acids, aldehydes, and ketones that can react with proteins to generate protein fragments [42]. In our study, two different bottles of PS80 were used between Phase 2 and Phase 3 studies. The quality of these two bottles of PS80 were tested by a reverse-phase HPLC method with charged aerosol detection. Results are presented in Fig. S6. Evidently the older bottle of PS80, which was used in the Phase 2 study, contained more degradation compared to the newer bottle of PS80 utilized in the Phase 3 study. We postulate the older bottles of PS80 with degraded PS80 contained higher levels of degradants that lead to protein fragmentation and provided less protection against aggregation, thus explaining the association between higher PS80 level and the increased fragmentation and aggregation in Phase 2. This association was not found in the Phase 3 study where a new bottle of PS80 was used.

In Phase 3, a significant increase in covalently linked aggregate was found in both new and control formulations, particularly under the 40°C/75%RH stress condition (Fig. 7b and c). The nature of these covalently linked aggregates remains unknown; however, the Fc domain contains disulfide bonds which could participate in intermolecular disulfide bond exchange to form covalent aggregates. We proposed that the partial unfolding of the tertiary structure during thermal stress exposed the internal hydrophobic residues that were responsible for the aggregate formation. Future studies to compare accelerated stability of LABL-Fc-ST and LABL-Fc-MOG_R5_ may elucidate whether the conjugated peptide is responsible for the instability of the LABL-Fc-MOG_R5_ protein. A significant fragmentation increase was seen in the control formulation group, particularly under 40°C/75%RH stress, by SE-UPLC, NR-CGE, and R-CGE (Fig. 6b and d). The nature of the fragmentation reaction is still unidentified, and the identity of these fragments will be characterized in our future studies. The observed acidic species may be due to deamidation of the PENNY peptide at pH of 7.2 in the control formulation and this reaction is less favorable at pH 5.3 in the new formulation (Fig. 6e).

## Conclusion

The final formulation effectively protects the LABL-Fc-MOG_R5_ protein from aggregation, fragmentation, and charge variants during freeze/thaw, mechanical stress, and 5°C storage. Based on thorough statistics-driven formulation development and stability assessment, the optimized formulation of the LABL-Fc-MOG_R5_ protein was at pH 5.3 in a solution containing 10 mM sodium acetate, 125 mM sucrose, 150 mM HBP-LB-BCD, and 0.025% PS80. Under the 25°C/60%RH condition, only a minor increase in particles was observed. In contrast, the control formulation showed significant degradation at the 40°C/75%RH condition. The inclusion of HP-βCD likely stabilized hydrophobic residues in the MOG peptide region, mitigating protein unfolding and aggregation. Collectively, this work establishes a robust formulation strategy for advancing the LABL-Fc-MOG_R5_ protein toward clinical development. In the future, we will evaluate the effects of this formulation on other Fc-BPI molecules (i.e. LABL-Fc-PLP, LABL-Fc-MBP). Finally, the role of myelin peptides on the stability of Fc-BPI molecules will be evaluated.

## Abbreviations

ADC antibody–drug conjugate

ADCC antibody-dependent cellular cytotoxicity

BPI bifunctional peptide inhibitor

EAE experimental autoimmune encephalomyelitis

Fc fragment crystallizable region of antibody

HP-β-CD hydroxypropyl-β-cyclodextrin

HPB-BCD hydroxypropyl-β-cyclodextrin (Biopharma grade)

HPB-LB-BCD lauroyl-modified hydroxypropyl-β-cyclodextrin

icIEF imaging capillary isoelectric focusing

IF-DSF intrinsic fluorescence differential scanning fluorimetry

IgG immunoglobulin G

LABL ICAM-1 ligand peptide

MBP myelin basic protein

MFI micro-flow imaging

MOG myelin oligodendrocyte glycoprotein

PBS phosphate-buffered saline

PLP proteolipid protein

PS80 polysorbate 80

SVP subvisible particle

## Supplementary Material

Supplemental_Information_tbag005

## Data Availability

The authors confirm that the data supporting the findings of this study are available within the article and its Supplementary Materials, or upon reasonable request to the corresponding author.

## Animal research statement

None declared.
