# Supplementary material for: Preventing physical and chemical degradation of the LABL-Fc-MOGR5, a bifunctional peptide inhibitor, with formulation development approaches
Source: Antib Ther. 2026 Feb 24;9(3):215–29. doi: 10.1093/abt/tbag005 (PMC13317743; doi:10.1093/abt/tbag005)
Supplement: Supplemental_Information_tbag005 [file supplemental_information_tbag005.docx]

**Supplemental Information**

**Initial Conjugation trial**

An initial conjugation trial was completed by mixing 50 mg of LABL-Fc-ST, 3.7 mg of sortase-A, 77 mg of MOG-R_5_ in 1X TBS, 6 mM CaCl_2_, in a total volume of 30 mL in 125 mL shake flask and incubate at 37°C for 24 hours. Reduced deglycosylated intact mass spectrometry was used to analyze the raw material (LABL-Fc-ST) and the conjugated product (Figure S1). Prior to conjugation, theoretical molecular weight after reduction and deglycosylation of LABL-Fc-ST was 27023 Da and the observe mass for the main peak was 27024 Da After conjugation, theoretical molecular weight after reduction and deglycosylation of LABL-Fc- MOG-R_5_ was 29391 Da and the observe mass for the main peak was 29391 Da. After conjugation, the conjugated product was purified by protein A chromatography, and the expected molecular weight was found in the final purified product. Thus, the conjugation trial was confirmed to be successful.


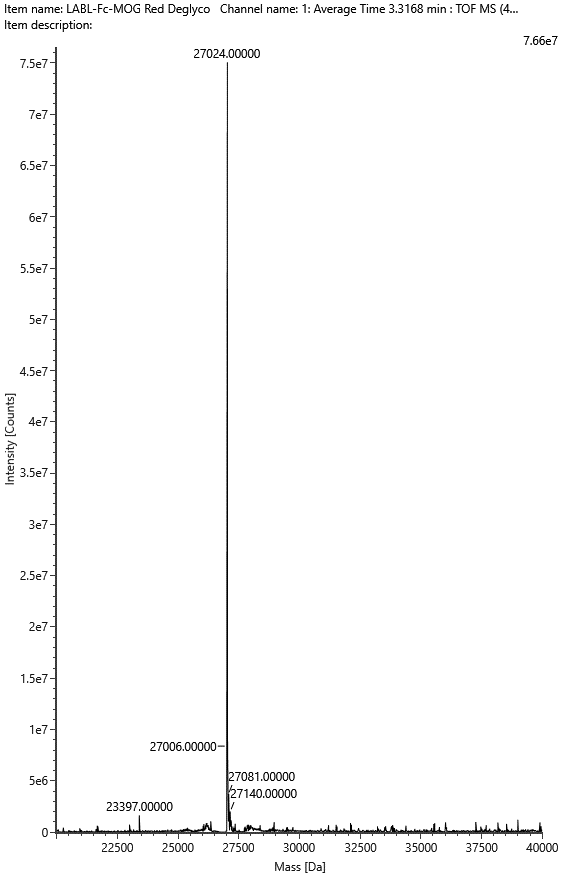

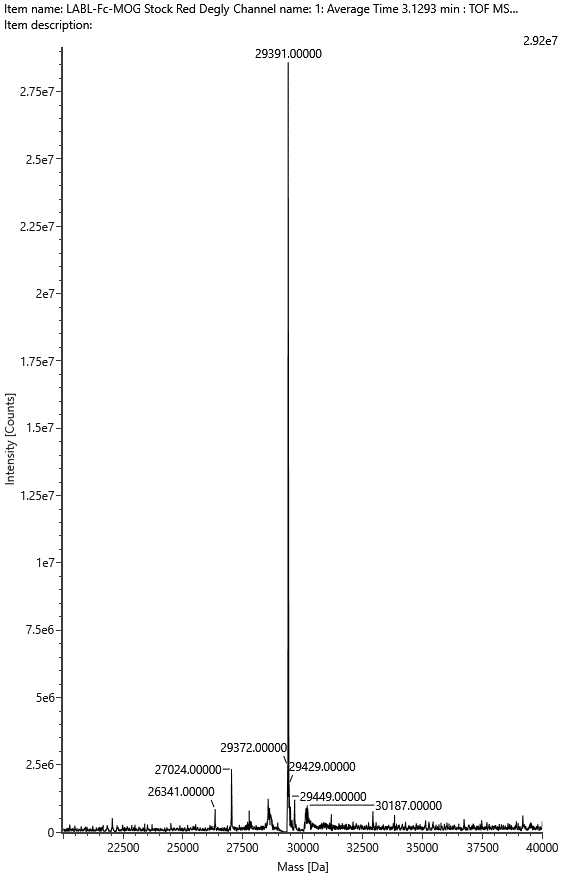

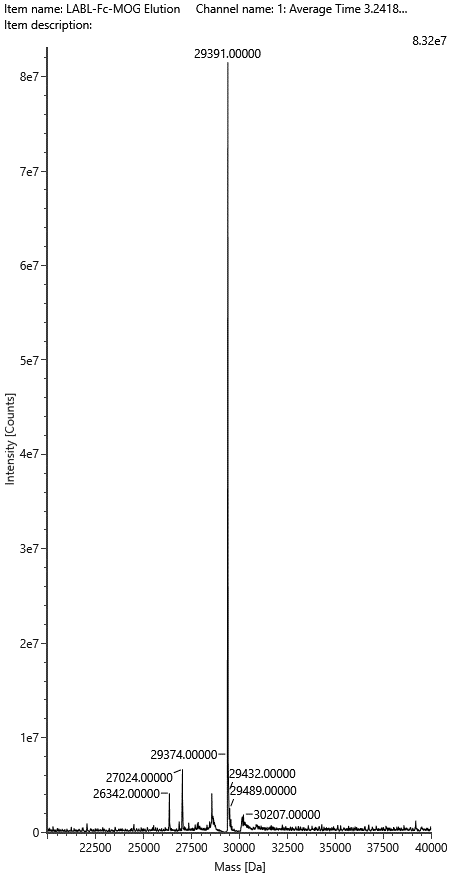


**C**

**B**

**A**

***Figure S1.*** Reduced and deglycosylated intact mass spectrometry results for **(A)** LABL-Fc-ST, **(B)** conjugation product LABL-Fc-MOGR5, and **(C)** purified conjugation product during initial trial conjugation.

**Conjugation scale-out**

Upon successful conjugation trial, the reaction was scaled out to three more conjugations to generate a total of 200 mg of conjugated product. The scaled-out conjugation products were combined and purified through protein A chromatography with elution at pH 3.6 and neutralized to pH 5.5 with 1 M Tris. The conjugated and purified product are tested with reduced deglycosylated intact mass spectrometry.


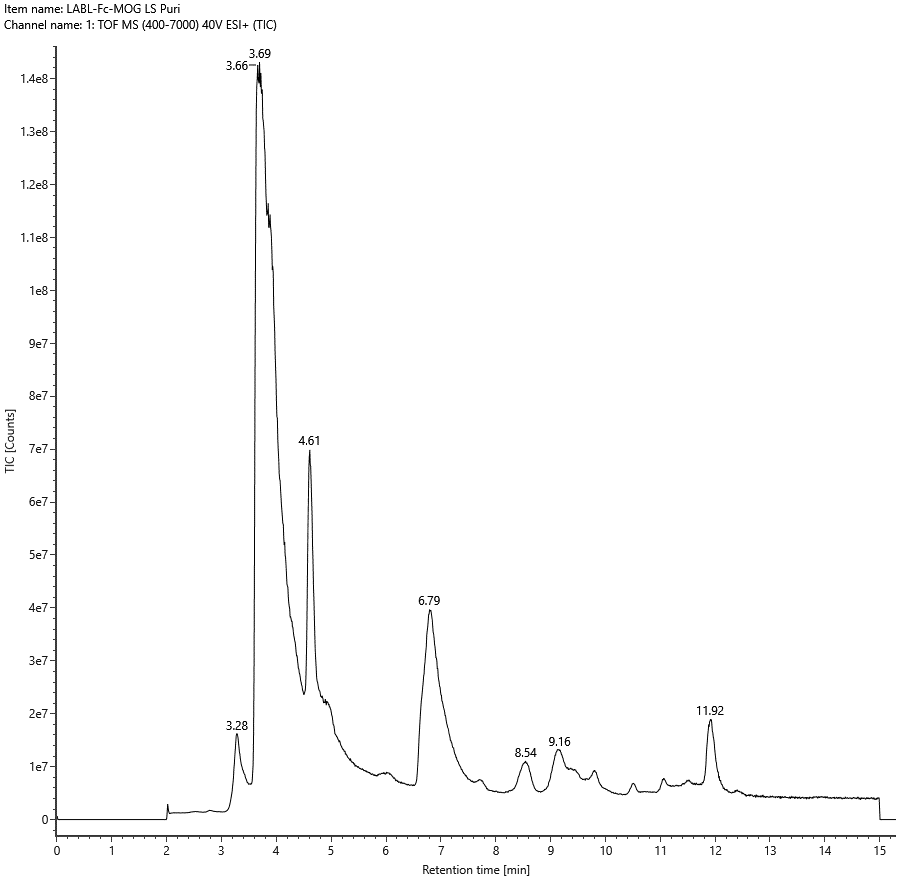

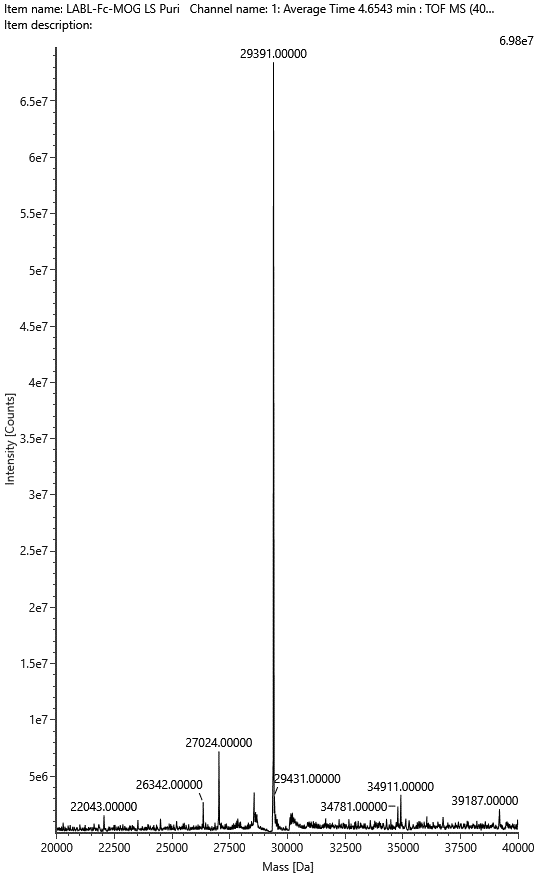


**B**

**A**

***Figure S2.*** Reduced and deglycosylated intact mass spectrometry results for **(A)** LABL-Fc- MOGR_5_ in UV chromatograph and **(B)** deconvoluted mass spectrogram.

***Figure S3.*** JMP analyses for T_onset_ for LABL-Fc-ST (left) and LABL-Fc-MOG_R5_ (right) in 96 formulations.


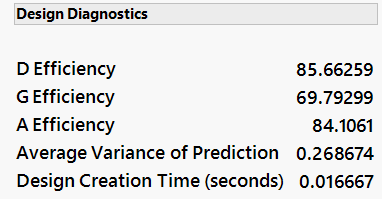

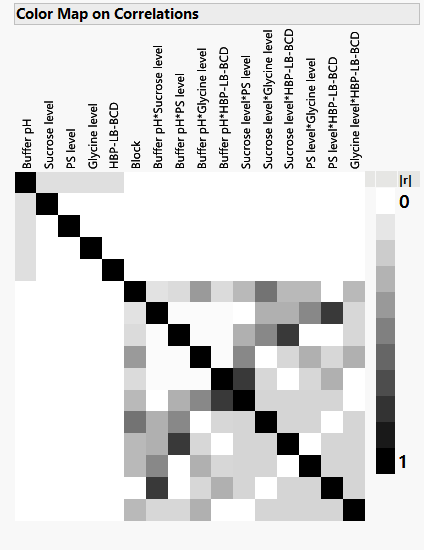


***Figure S4*.** Design evaluation for DSD formulation optimization study. Correlations mapping is presented on the left and the design diagnostics are shown on the right.

**Figure S5.** DoE formulation screening results averaged with multiple sample preparations and across different timepoints. **(A)** %HMW by SE-UPLC (A) and **(B)** %LMW by SE-UPLC are presented with three sample preparations with four timepoints for N=12. %Aggregates by NR-CGE and %Fragments by NR-CGE are presented with two sample preparations with four timepoints for N=8.


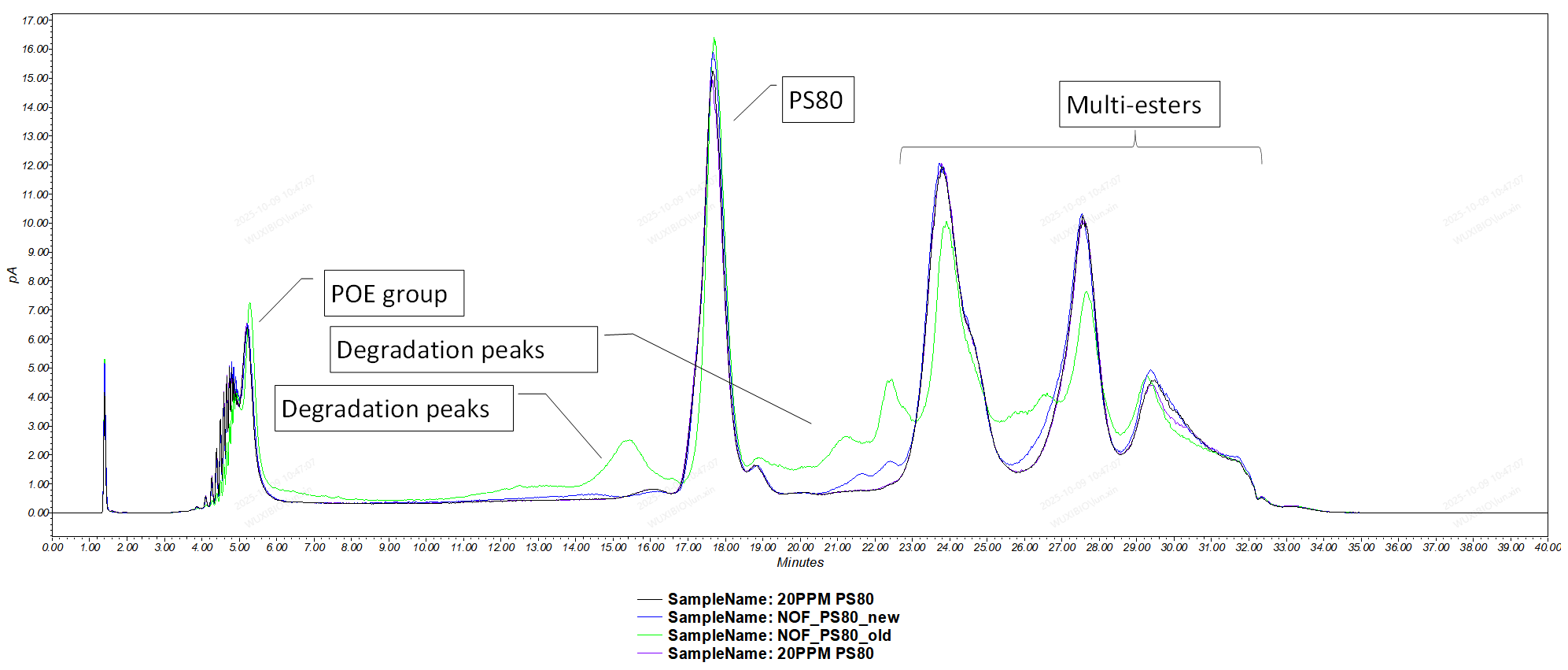


**Figure S6**. Polysorbate degradation investigation using RP-HPLC charged aerosol detection. Two PS80 products were tested, both produced by NOF America (White Plains, NY). NOF_PS80_old (green trace) was the product used in the phase 2 study, and NOF_PS80_new (blue trace) was the product used in the phase 3 study. Bracketing injections of Croda [Super Refined™ Polysorbate 80 (black and purple traces) were added to the overlay. Peaks groups that were previously identified by LC-MS (proprietary data) were labelled.](https://www.crodapharma.com/en-gb/product-finder/product/681-super_1_refined_1_polysorbate_1_80)
